# Beam shape effect on enhanced laser wakefield acceleration of electrons driven by 10-fs mJ-class pulses

**DOI:** 10.1038/s41598-026-41516-0

**Published:** 2026-02-26

**Authors:** Mehdi Abedi-Varaki, Vidmantas Tomkus, Valdas Girdauskas, Gediminas Račiukaitis

**Affiliations:** 1 FTMC - Center for Physical Sciences and Technology, Savanoriu Ave. 231, Vilnius, LT-2300 Lithuania; 2 Vytautas Magnus University, K. Donelaicio St. 58, Kaunas, LT-44248 Lithuania

**Keywords:** Laser wakefield acceleration, Fourier–Bessel particle-in-cell simulation, Ionisation injection, Pulse evolution, High-repetition-rate lasers, Engineering, Optics and photonics, Physics

## Abstract

Laser wakefield acceleration (LWFA) of electrons was investigated using quasi-3D Fourier–Bessel Particle-In-Cell (FBPIC) simulations to optimise the interplay between laser beam structure and plasma target parameters. Gaussian (G) and Bessel–Gauss (BG) beams driven by a 40 mJ, 10 fs laser pulse were compared, representing few-cycle mJ-class (10–100 mJ) laser systems, across a range of the normalised vector potentials *a*_*0*_ = 1.75–5.6. The results show that, for a fixed laser system with 40 mJ pulse energy and 10 fs duration, BG beams extend the effective acceleration length and improve electron energy gain by 20–27% relative to G drivers, despite variations in _*0*_ arising from different focal geometries. The extended focal zone of BG beams mitigates diffraction losses, while ionisation injection from a 1% nitrogen admixture produces stable injection over a distance of ~ 100 μm at *a*_*0*_​=2.0. However, laser pulse evolution leads to an intensity drop by a factor of 2–2.5 over 300–500 μm, ultimately limiting the maximum acceleration length. These findings confirm that BG beams offer a robust pathway to enhance LWFA performance at sub-100 mJ laser energies, with direct implications for compact high-repetition-rate plasma accelerators.

## Introduction

Laser wakefield electron acceleration (LWFA) was first analysed using Particle-In-Cell (PIC) simulations by Tajima and Dawson in 1979^1^. The LWFA can provide accelerating fields more than three orders of magnitude higher than those achievable in conventional RF-based particle accelerators^2^. The energies of LWFA accelerated electrons up to 10 GeV have been reported with PW-class lasers^3^.

The maximum energy of accelerated electrons at fixed pulse energy and duration is limited mainly by the dephasing length (*L*_*d*_) and the laser pump depletion length(*L*_*dep*_)^4^. *L*_*d*_ is the distance over which electrons outrun the plasma wave and enter a decelerating phase^5,6^. *L*_*dep*_ is the length over which half of the laser energy is transferred to the plasma wave^7^. The group velocity of the light pulse in plasma is lower than the velocity of the electron bunch. The longitudinal plasma field accelerates electrons, increasing their energy for the acceleration lengths (*L*_*acc*_) shorter than *L*_*d*_. For *L*_*acc*_*> L*_*d*_, the longitudinal accelerating field within the plasma bubble changes its sign, and the bunch starts to decelerate. The electron bunch energy is maximum when *L*_*acc*_ reaches *L*_*d*_^8^.

Even though ultra-short, multi-TW-scale lasers easily exceed pulse energy and intensity requirements for LWFA, the dephasing and depletion restrictions limit the electron peak energies in LWFA from hundreds of MeV to several GeV^9,10^. To mitigate these limitations and reduce the energy dispersion and divergence of accelerated electrons, a variety of approaches have been developed, including preformed plasma channels^11^, gas-filled capillary discharge waveguides^12^, laser self-channelling^13^. Electron self-injection has been demonstrated in numerous LWFA experiments and enables electron trapping without additional experimental complexity^14,15^. Nevertheless, this mechanism is highly sensitive to nonlinear laser evolution and plasma conditions, often resulting in limited stability and broad energy spectra.

Producing high-quality electron bunches through this mechanism in a stable and reproducible manner is challenging, as it is strongly affected by nonlinear effects such as laser self-focusing, spectral broadening, and self-steepening^16–18^. In addition, once the plasma wave reaches the wave-breaking limit, further electron injection from the plasma becomes impossible^19^. Among the various techniques, ionisation injection is the most straightforward and efficient method to inject electrons into the wakefield. In this scheme, inner-shell electrons of high-Z atoms are injected into the wake bubble at a specific phase^20–22^. Although this method allows injection at lower laser intensities and densities, continuous injection can increase the energy spread. Thus, ionisation injection often leads to a large energy spread.

Various schemes have been proposed to reduce the injection distance and the energy spread, such as using two-colour lasers for controlling injection^23^, employing two gas cells to separate the electron injection and electron acceleration^24–28^, or self-truncated ionisation injection (STII)^29^. Recently, Malik et al.^30^ investigated LWFA driven by structured laser beams and demonstrated that tailoring the transverse laser profile significantly modifies the plasma wake structure and electron injection dynamics, leading to distinct acceleration characteristics compared to conventional G drivers. Using PIC simulations, Zeng et al.^29^. demonstrated STII, in which electron injection occurs only in the front region of a He–N_2_ mixed gas target over a distance of a few hundred micrometres. To generate high-quality electron beams using the STII method, several stringent conditions should be satisfied. These include maintaining the peak laser intensity below the self-injection threshold to suppress uncontrolled trapping^31,32^, limiting the effective injection length to avoid multiple injection events, and ensuring stable laser propagation to prevent local intensity hotspots that degrade beam quality^33^. However, it should be noted that in these schemes, a narrow energy spread is typically obtained at the cost of a lower electron beam charge.

Among the most critical factors governing electron acceleration in LWFA, the nonlinear spatio-temporal evolution of the driving laser pulse plays a central role. Processes such as relativistic self-focusing, pulse-front etching, spectral red-shifting, and pump depletion strongly modify the wakefield structure and injection dynamics. Previous studies have shown that tailoring the spatial and temporal properties of the laser pulse can significantly influence acceleration performance^34,35^. However, systematic comparisons of Gaussian (G)and Bessel–Gaussian (BG) beams for few-cycle, mJ-class pulses remain limited, particularly in the tight-focusing regime where nonlinear effects occur on sub-millimetre scales. In this low-energy, tight-focusing regime, nonlinear laser evolution and depletion occur on sub-millimeter scales, making the interplay between beam structure and acceleration distance especially critical.

Emerging kilohertz laser systems delivering few-cycle pulses with energies of several tens of millijoules enable compact and cost-effective LWFA platforms^36–38^. These systems are attractive for applications requiring high stability and high average flux, such as ultrafast diagnostics, material science, and radiotherapy. However, to drive electrons in a self-guiding bubble regime with a laser of limited pulse energy requires relatively high plasma densities of *n*_*e*_=3–5 × 10^19^ cm^− 3^ and tighter focusing of the laser beam to a diameter of 3–5 μm. It leads to shorter Rayleigh and dephasing distances and limits the energy of accelerated electrons.

In this paper, we investigate LWFA driven by G and BG laser beams using quasi-3D Fourier–Bessel particle-in-cell (FBPIC) simulations. All simulations are performed for a fixed laser system with 40 mJ pulse energy and 10 fs duration, representative of emerging kHz-class sources. Within this constraint, the laser focal geometry and consequently the peak normalised vector potential _*0*_ are varied to optimise acceleration. We systematically compare laser evolution, wakefield structure, ionisation injection, and electron beam properties, with particular emphasis on the role of extended focal depth in determining the effective acceleration length and energy gain.

This paper is organised as follows. In Sect. 2, the methods of PIC simulation are described. Section 3 presents the simulation results of electron bunch parameters accelerated using G and BG beams. In Sect. 4, the ionisation injection effects are analysed, and in Sect. 5, the pulse evolution effects of G and BG beams depending on laser intensity and plasma density are discussed. Finally, Sect. 6 presents the conclusions of the research.

## Methods

In this paper, the simulation of the LWFA of electrons driven by G and BG beams was investigated using the quasi-3D PIC code FBPIC^39^. The code employs an azimuthal modal decomposition of the electromagnetic fields, enabling efficient simulation of fully 3D laser–plasma interactions while retaining the essential physics captured by standard 3D PIC codes, but at a significantly reduced computational cost. This approach is particularly well suited for G and BG laser drivers, whose near-cylindrical symmetry allows accurate representation using a limited number of azimuthal modes, while fully accounting for laser diffraction, relativistic self-focusing, self-modulation, ionisation injection, and plasma wake evolution.

Electron acceleration simulations were carried out for various normalised vector potentials and beam waist radii of both G and BG pulses. In all cases, the laser pulses had a Gaussian temporal envelope. For the G pulse, a simulation window size of *r × z* = 60 × 30 µm^2^ was chosen to span at least two plasma wave periods along the longitudinal *z*-axis and to exceed at least five times the initial G beam waist along the radial coordinate *r*. This choice ensures that the diffracting laser beam and the associated plasma wake remain fully contained within the simulation window throughout the propagation distance of interest.

For the BG pulse, the simulation window size was increased to *r × z* = 250 × 30 µm^2^. Unlike G beams, BG beams possess a central core surrounded by concentric side lobes that extend over a much larger radial distance. These side lobes play a crucial physical role by continuously replenishing energy into the central lobe, thereby sustaining the on-axis intensity over an extended propagation length. A substantially larger radial domain is therefore required to accurately capture this energy-replenishment mechanism and to avoid artificial boundary effects that could distort the laser beam evolution and wakefield structure. Along the transverse coordinate *r*, the window size was chosen to be approximately 50 times larger than the width of the central BG peak, ensuring minimal influence of the radial boundaries on the laser–plasma interaction.

Open boundary conditions were applied at the edges of the simulation window to further suppress unphysical reflections. The coordinate grids were set to *N*_*r*_ *× N*_*z*_ = 300 × 600 for the G pulse and *N*_*r*_ *× N*_*z*_ = 600 × 600 for the BG pulse. The different values of *N*_*r*_ reflect the different radial extents of the two beam types and were chosen so that the radial grid spacing *Δr* remains comparable in both cases, ensuring similar numerical resolution of the laser transverse structure, plasma skin depth, and wakefield gradients. Using the same *N*_*r*_ ​for both beams would either under-resolve the extended radial structure of the BG beam or unnecessarily over-resolve the G case, leading to inefficient computation without improving physical accuracy. The longitudinal grid resolution *N*_*z*_ was kept identical to ensure a consistent treatment of the laser pulse temporal evolution, plasma wave phase velocity, dephasing, and pump depletion in the comparative analysis.

Both G and BG cases were simulated using (2, 2, 3) macro-particles per cell and three azimuthal modes (*m* = 3), which were found to be sufficient for accurately capturing the relevant laser–plasma dynamics while maintaining computational efficiency.

The LWFA simulations were conducted in hydrogen plasma with a linear density ramp of 10 μm, increasing from vacuum to the target plasma electron density *n*_e_ at the beginning of the laser propagation region. In the remaining simulation zone, the plasma density was kept constant at *n*_*e*_. To initiate ionisation injection, 1% of nitrogen atoms relative to the hydrogen density were introduced at the start of the laser beam propagation. The nitrogen concentration increased linearly over a 10 μm ramp and then remained constant over a distance *L*_*N*_. Beyond *z >* 10 *µm + L*_*N*_, the nitrogen density was set to zero. This configuration enables controlled ionisation injection while avoiding continuous trapping at later propagation stages.

### PIC simulation of LWFA using G beam

In the analysis of LWFA using a G beam, the G temporal and spatial profile of the envelope of the normalised vector potential *a*_*0*_ of a linearly polarised laser pulse has been used^35^:

1 $$a(r,\eta )={a_0}\frac{{{w_0}}}{{{w_G}}}\exp \left( {ik\zeta - \frac{{{r^2}}}{{w_{G}^{2}}} - \frac{{{\eta ^2}}}{{{{(c\tau )}^2}}}} \right)$$

where, *k = 2π/λ*, ${w_G}={w_0}\sqrt {1+{{{\xi ^2}} \mathord{\left/ {\vphantom {{{\xi ^2}} {z_{R}^{2}}}} \right. \kern-0pt} {z_{R}^{2}}}}$, *w*_*0*_ is the radius at the beam waist,${z_R}={{\pi w_{0}^{2}} \mathord{\left/ {\vphantom {{\pi w_{0}^{2}} \lambda }} \right. \kern-0pt} \lambda }$is the Rayleigh length, *ζ = z ˗ z*_*f*_ is the relative position of the focal point coordinate of the G beam *z*_*f*_, *η = z˗ ct ˗ z*_*c*_ is the longitudinal coordinate of the moving simulation window, *z*_*c*_ is the coordinate of the pulse peak, *r* is the radial coordinate, and *λ* and *τ* are the laser wavelength and pulse duration. In the analysis, the laser pulse energy *E*_*L*_=40 mJ, pulse duration at the Full Width Half Maximum (FWHM) of the intensity *τ*_*0.5*_ = 10 fs, and laser wavelength *λ* = 900 nm were chosen.

The LWFA simulations were carried out for various *a*_*0*_ values, corresponding to the laser intensity *I*_*0*_ *= 2E*_*L*_*/τ*_*0.5*_*/πw*_*0*_^*2*^, achievable by focusing the G beam to a spot area equal to *πw*_*0*_^*2*^*/*2. The corresponding electric field of the laser pulse was recalculated as,${E_0}=\sqrt {2{I_0}/c{\varepsilon _0}}$, and the normalised vector potential as *a*_*0*_*=eE*_*0*_*/m*_*e*_*c*^*2*^*k*.

Initially, the required plasma electron density *n*_*e*_ for the acceleration in bubble regime, corresponding to the specific *a*_*0*_ parameter and laser beam waist radius *w*_*0*_, as well as the Rayleigh length *z*_*R*_, dephasing length *L*_*d*_, laser pulse energy depletion length *L*_*dep*_, and ratio of laser peak power *P*_0_ and the critical relativistic self-focusing power *P*_*cr*_ were calculated using analytical expressions^10^.

The blow-out radius *R*_*b*_ of the plasma bubble should match the laser spot size,$\:\:{w}_{0}\simeq\:{R}_{b}=\frac{{\lambda\:}_{p}\sqrt{{a}_{0}}}{\pi\:}$where *λ*_*p*_ is the plasma wavelength, and for the pulse duration *τ*_0.5_, the condition *w*_*0*_ *> cτ*_*0.5*_ should be satisfied^40,41^. The plasma electron density *n*_*e*_ was calculated as${n_e}={{{4{\pi ^2}{c^2}{m_e}{\varepsilon _0}} \mathord{\left/ {\vphantom {{4{\pi ^2}{c^2}{m_e}{\varepsilon _0}} {\left( {{\lambda _p}e} \right)}}} \right. \kern-0pt} {\left( {{\lambda _p}e} \right)}}^2}$. The dephasing length *L*_*d*_ over the bubble radius *R*_*b*_ was estimated as$\:\:{L}_{d}\simeq\:\frac{4\sqrt{{a}_{0}}{\lambda\:}_{p}^{3}}{3\pi\:{\lambda\:}^{2}}$, and the pump depletion length $\:{L}_{dep}\simeq\:\frac{{\lambda\:}_{p}^{2}c{\tau\:}_{0.5}}{{\lambda\:}^{2}}$. The laser peak power *P*_*0*_ and the critical relativistic self-focusing power *P*_*cr*_ were calculated as $\:{P}_{0}\left[GW\right]\simeq\:21.5{\left(\frac{{a}_{0}{R}_{b}}{\lambda\:}\right)}^{2}$and$\:{P}_{cr}\left[GW\right]\simeq\:17{\left(\frac{{\lambda\:}_{p}}{\lambda\:}\right)}^{2}$, correspondingly^4,42^.

In the computational analysis, the analytically calculated plasma density *n*_*e*_ was optimised using the FBPIC simulation, and the optimal plasma density *n*_*opt*,_ corresponding to the maximum energy of LWFA-accelerated electrons, was chosen. The plasma density values for various *a*_*0*_ and beam waist radius *w*_*0*_ parameters of the G beam used in the simulation are presented in Table.

**Table 1:** The optimal plasma density and corresponding Rayleigh, dephasing, depletion lengths, and the ratio of the laser peak power and the critical relativistic self-focusing power for various $\:{a}_{0}$ normalised vector potential and waist radius *w*_*0*_ parameters of Gaussian beams (G_1_ - G_5_)with the pulse duration *τ*_*0.5*_ = 10 fs and energy of 40 mJ.

Beam shape	a_0_	w_0,_ µm	*n*_e_, ⋅10^19^ cm^− 3^	z_*R*,_ µm	*n*_opt_, ⋅10^19^ cm^− 3^	L_d,_ µm	L_dep,_ µm	*P*_0_/*P*_cr_
G_1_	4.1	3.0	5.2	31	2.2	369	183	8.6
G_2_	3.0	4.0	2.1	56	2.0	363	201	3.3
G_3_	2.5	5.0	1.1	87	1.6	465	252	2
G_4_	2.0	6.0	0.63	126	1.3	568	310	1
G_5_	1.75	7.0	0.39	171	1.3	531	310	0.7

It can be seen from Table 1 that the Rayleigh length *z*_*R*_ drops with a decrease in the beam waist. The maximum acceleration distances *L*_*d*_ and *L*_*dep*_ become shorter as plasma density increases.

For *a*_*0*_ = 3, the optimal density *n*_*opt*_ corresponds quite well to the analytically calculated *n*_*e*_. For a density below the optimal density *n*_*e*_, the energy of accelerated electrons decreases due to the lower longitudinal accelerating electric field in the plasma. For a density higher than the optimal density *n*_*e*_, the energy of accelerated electrons is limited by the shortening of the *L*_*d*_ and *L*_*dep*_ lengths. However, for *a*_*0*_ = 1.75 and *a*_*0*_ = 2, the *n*_*opt*_ exceeds the analytically calculated *n*_*e*_. For lower *a*_*0*_ and *n*_*e*_ values, the ratio of the laser peak power to the critical relativistic self-focusing power, *P*_0_*/P*_*cr*_, drops below 2. A higher plasma density is required to maintain the effective acceleration distance. For *a*_*0*_ = 4, *n*_*opt*_ is lower than the analytically calculated value *n*_*e*_. For *a*_*0*_ > 4 and short 10 fs pulses, the acceleration distance is limited by the effects related to the laser energy loss rather than by the dephasing length^4^.

### PIC simulation of LWFA using BG beam

As discussed in Sect. 2.1, the LWFA acceleration using a G beam, the maximum acceleration distance and electron energy are limited by the laser pulse energy, electron bunch dephasing and laser pulse depletion. In this paper, to increase the energy of accelerated electrons, we propose to use a BG laser beam formed by an axiparabola or a reflecting axicon^43,44^, which, compared to a G beam, ensures a longer acceleration distance. A lower optimal plasma electron density, *n*_*e*_, results in larger *L*_*d*_ and *L*_*dep*_ lengths.

**Fig. 1:**
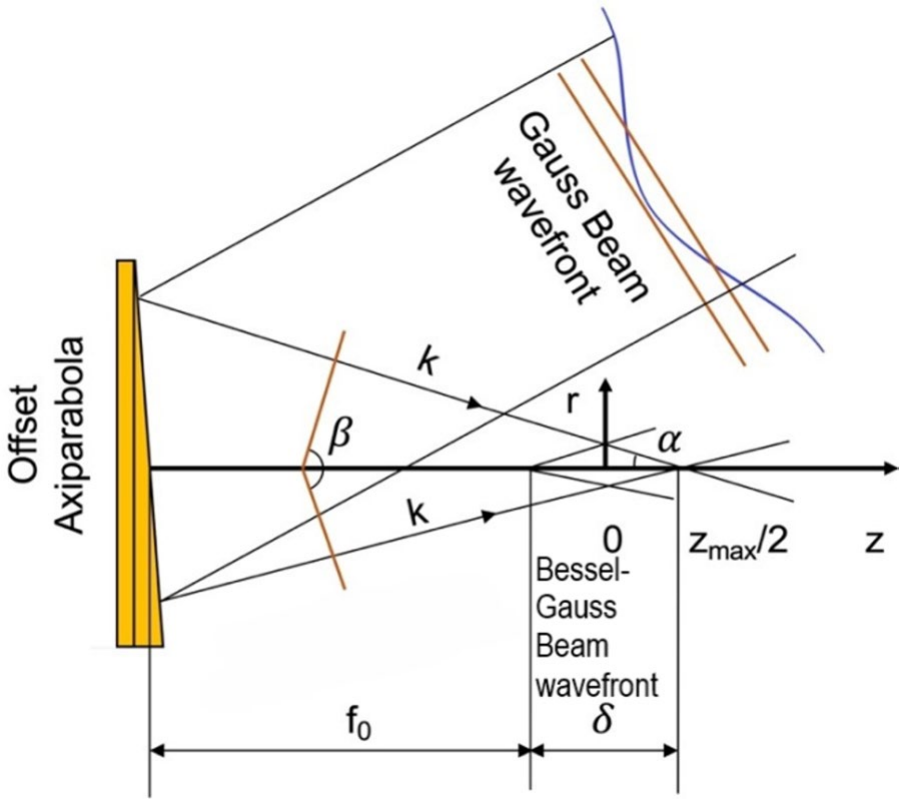
The diagram of the formation of a BG beam with extended focal zone length *δ* using an offset axiparabola illuminated by a G beam. The LWFA acceleration simulation was started at the maximum intensity point of the laser pulse, corresponding to *z*_*max*_*/*2.

An essential feature of the axiparabola is the ability to vary independently the intensity of the focused laser pulse, *I*_*0*_*=kE*_*0*_*/δτ*_*0.5*_, where *δ* is the focal zone length and the beam radius in the focal zone ${r_o}=0.77\lambda N\sqrt {{\delta \mathord{\left/ {\vphantom {\delta z}} \right. \kern-0pt} z}}$, where *N = f*_*0*_*/D* is the f-number of the axiparabola. *f*_*0*_ and *D* are the focal distance and diameter of the axiparabola, correspondingly. Independent variation of the focal zone length *δ* and the Bessel beam radius allows optimisation of the parameters of accelerated electrons, given the available laser pulse energy and duration. In the analysis of laser wakefield acceleration using a BG laser beam, the solution of the paraxial wave equation of the normalised vector potential *a*_*0*_ of the linearly polarised BG beam in the FBPIC simulation was used^45^:

2 $$a(r,\eta )={a_0}\frac{{{w_0}}}{{{w_G}}}J_{0}^{2}\left( {\frac{{2.4048r}}{{{w_b}(1+\frac{\zeta }{{{z_R}}})}}} \right)\exp \left( { - \frac{{{r^2} - {\eta ^2}{{\sin }^2}{\alpha _0}}}{{w_{G}^{2}}} - i\left( {k\eta \left( {1 - \frac{{{{\sin }^2}{\alpha _0}}}{2}} \right) - \frac{\pi }{2}} \right) - {{\left( {\frac{\eta }{{c\tau }}} \right)}^2}} \right)$$

where *J*_*0*_ is the zero^th^-order Bessel function of the first type, *w*_*b*_=2.4048/*k* s*inα*_*0*_ is the Bessel beam radius at the first zero of the Bessel function *J*_*0*_. The quasi-non-diffracting beam does not have a uniform intensity distribution along its propagation axis and exists only over a limited range *z*_*max*_*=w*_*0*_ *cosα*_*0*_*/sinα*_*0*_, where *α*_*0*_ is the intersection angle of the reflected beams of the axiparabola with the propagation axis of the G beam, and *w*_*0*_ is the waist radius of the illuminating G beam^46^. The diagram of the formation of a BG beam with extended focal zone length *δ* using an offset axiparabola illuminated by a G beam is depicted in Fig. 1.

The formation of a BG beam with an extended focal zone length *δ* can be achieved using an axiparabola mirror illuminated by a Gaussian beam. The axiparabola reshapes the incoming Gaussian pulse into a BG beam that maintains a quasi-nondiffracting core over long distances, while a Gaussian beam diverges within its characteristic Rayleigh length. Physically, this allows the laser intensity and thus the ponderomotive forces driving the plasma bubble to remain effective over an extended acceleration length. This is crucial in LWFA, since the maximum electron energy is limited by dephasing and laser depletion.

Extending the effective focal region delays the on-axis depletion of the driving field by continuously replenishing the central intensity through radial energy flow from the surrounding rings of the BG beam. While the total laser energy is still depleted through wake excitation, this redistribution sustains the effective driver amplitude over a longer distance, thereby enabling higher electron energy gain. The initial electric field *E*_*0*_ for various *α*_*0*_ angles was normalised using the analytical relation of axiparabola intensity *I*_*0*_ *= kE*_*L*_*⁄δτ*_*0.5*_ for *δ = z*_*max*_. At the beginning of LWFA, *E*_*0*_ corresponds to maximal values *a*_*0*_ at a distance ~*z*_*max*_*/2* approximately while illuminating the axiparabola with a G beam with *a*_*0*_ = 2, intensity *I*_*0*_ *= E*_*L*_*/τ*_*0.5*_*/(πw*_*0*_^*2*^*/2)* and the waist radius of the G beam *w*_*0*_ = 6.1 μm.

The energy *E*_*L*_ = 40 mJ and pulse duration *τ*_*0.5*_ = 10 fs were chosen to match the laser pulse parameters of the G beam, as described in Sect. 2.1. The values *a*_*0*_ = 2 and *ω*_*0*_ = 6.1 μm were selected based on the available pulse energy and the maximum waist radius, resulting in the longest Rayleigh distance and minimal *a*_*0*,_ still ensuring effective ionisation of nitrogen to *N*^*+ 5*^, injection of electrons into the bubble and their acceleration in the bubble propagation regime. The maximum normalised vector potential *a*_*0max*,_ maximum propagation distance *z*_*max*_, Bessel beam radius *w*_*b*_, and optimal plasma density of the BG beams used in the simulation for various angles *α*_*0*_ are presented in Table 2.

**Table 2:** The maximal normalised vector potential *a*_*0max*_, waist radius *w*_*b*_, maximal propagation distance *z*_*max*_, and corresponding optimal plasma density, Rayleigh, dephasing, depletion lengths, and the ratio of the laser peak power and the critical relativistic self-focusing power of Bessel-Gauss beams (BG_01_-BG_06_) for various angles *α*_*0*_ and f-numbers *N* illuminated by a G beam with normalised vector potential *a*_*0*_ = 2.0, pulse energy 40 mJ, waist radius *w*_*0*_ = 6.1 μm and pulse duration *τ*_*0.5*_ = 10 fs.

Beam shape	α_0_, deg	*N*	a_0max_	w_b_, µm	z_max_, µm	*n*_opt_, ×10^19^ cm^− 3^	w_0_, µm	z_*R*_, µm	L_d_, µm	L_dep_, µm	*P*_0_/*P*_cr_
BG_01_	4.6	4.3	5.4	3.0	75	2.6	6	126	334	156	20
BG_02_	3.5	7.2	4.7	5.0	100	2.4	6	126	347	166	13
BG_03_	2.5	10.1	4.1	7.0	140	1.3	6	126	813	310	8.6
BG_04_	1.7	14.4	3.3	10.0	200	1.3	6	126	729	310	4.5
BG_05_	1.2	21.7	2.7	15.0	300	1.3	6	126	660	310	2.5
BG_06_	0.7	43.3	2.1	30.0	500	1.3	6	126	582	310	1.1

**Fig. 2:**
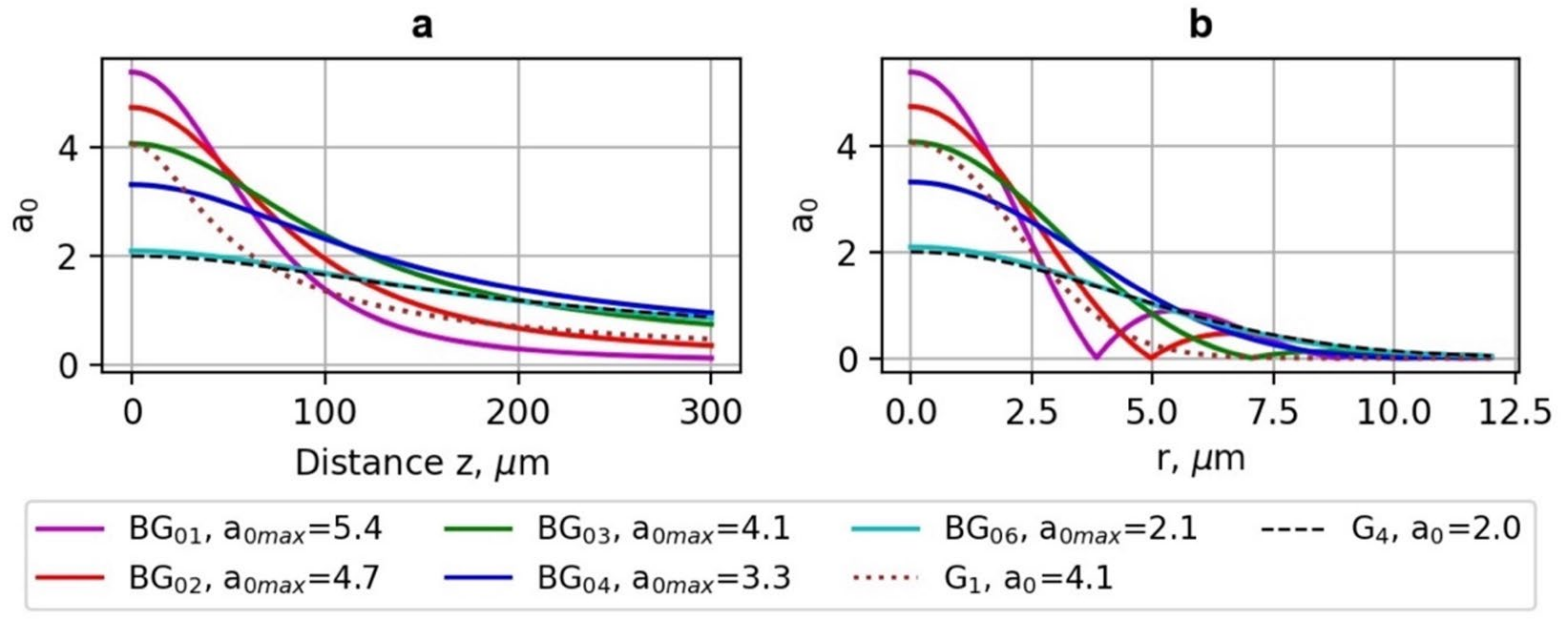
Dependence of the normalised vector potential *a*_*0*_ on the propagation distance *z* in vacuum (**a**) and radial coordinate *r* at *z* = 0 μm (**b**) of Bessel-Gauss (BG_01_-BG_06_) and Gaussian (G_1_, G_4_) beams.

The dependence of the normalised vector potential *a*_*0*_ on the propagation distance *z* in vacuum (a) and radial coordinate *r* at *z* = 0 μm (b) of the Bessel-Gauss (BG_01_-BG_06_), and Gaussian (G_1_, G_4_) beams is shown in Fig. 2.

## LWFA electron energy using G and BG beams

**Fig. 3:**
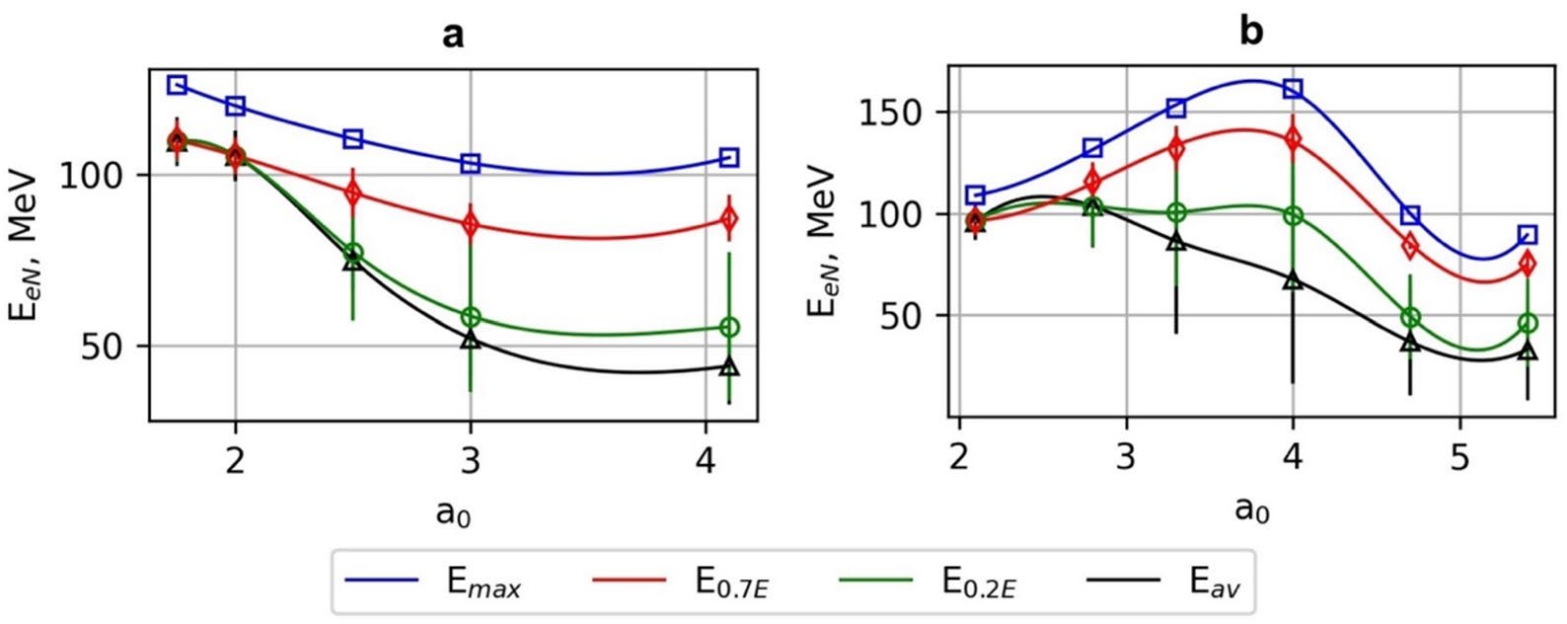
The dependence of maximal energy *E*_*max*_, average energies *E*_*0.7E*_, *E*_*0.2E*_, *E*_*av*_ of electrons accelerated using Gaussian beams (G_1_-G_5_) (**a**), and (**b**) Bessel-Gauss beams (BG_01_-BG_06_) on the normalised vector potential *a*_*0*_ and corresponding waist radius *ω*_*0*_ and *ω*_*b*_ parameters. The laser pulse energy is 40 mJ, and the pulse duration is *τ*_*0.5*_ = 10 fs.

**Fig. 4:**
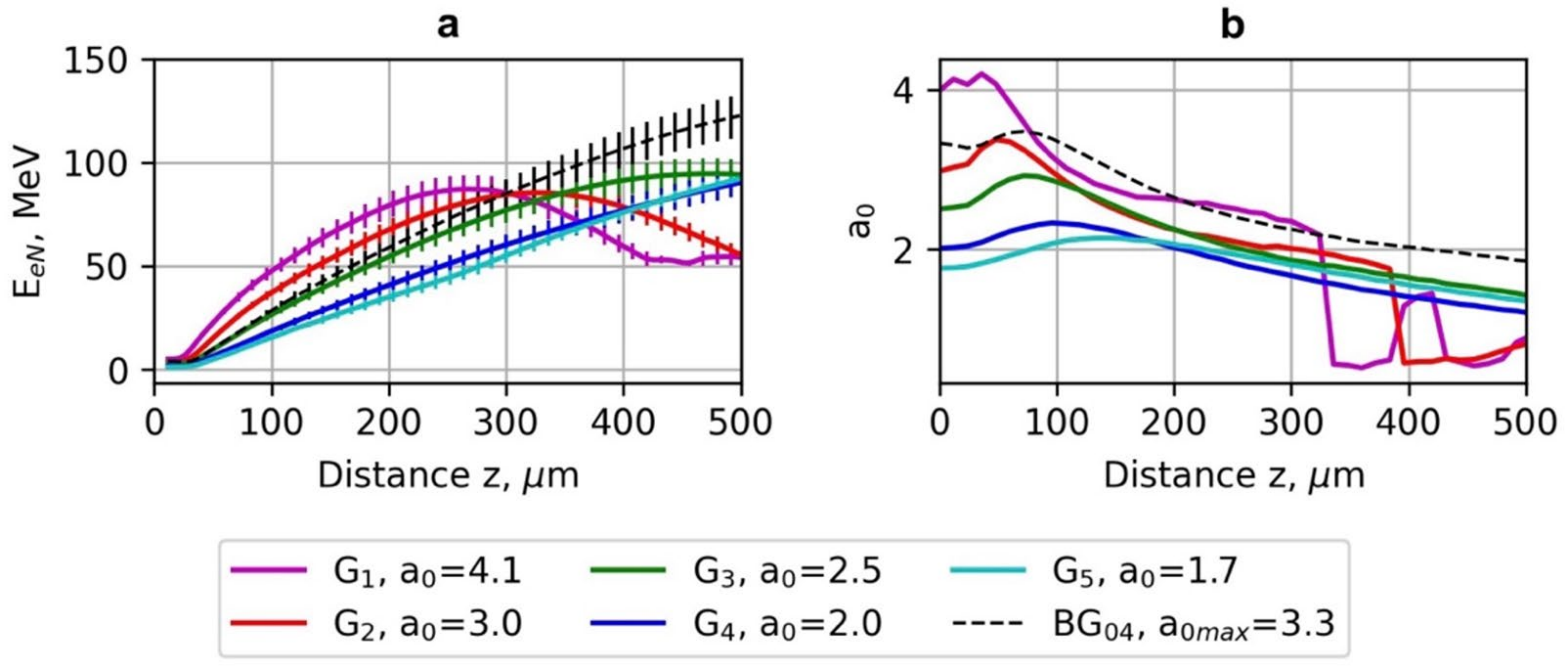
The dependence of average energy *E*_*0.7E*_ of electrons accelerated using the Gaussian (G_1_-G_5_) beams and Bessel-Gauss BG_04_ beam (a) and normalised vector potential *a*_*0*_ (b) on acceleration distance *z*. The laser pulse energy is 40 mJ, and the pulse duration is *τ*_*0.5*_ = 10 fs.

**Fig. 5:**
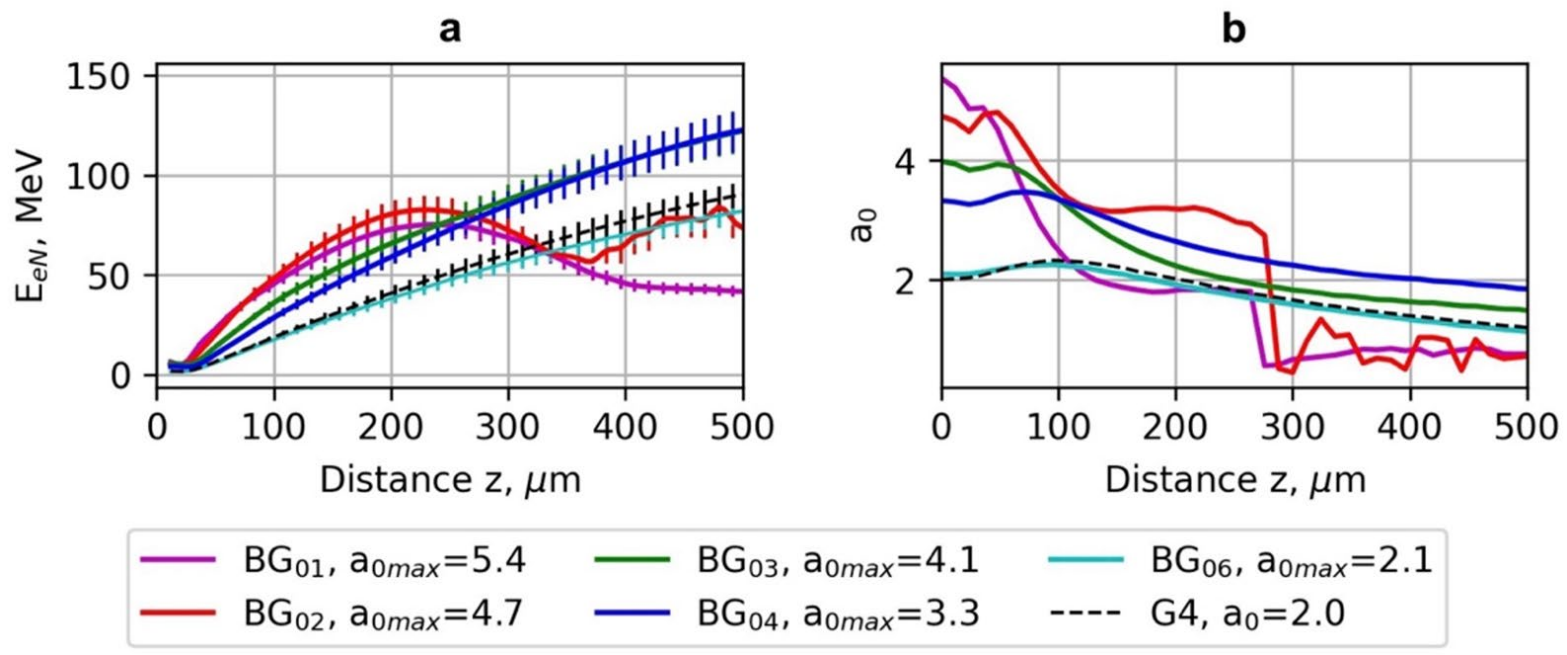
The dependence of average energy *E*_*0.7E*_ of electrons accelerated using the Bessel-Gauss (BG_01_-BG_06_) beams and the Gaussian beam (G_4_) (a) and normalised vector potential *a*_*0*_ (b) on acceleration distance *z*. The laser pulse energy is 40 mJ, and the pulse duration is *τ*_*0.5*_ = 10 fs.

The results of PIC simulation of the energy of LWFA of electrons carried out using the Gaussian beams (G_1_-G_5_) and Bessel-Gauss beams (BG_01_-BG_06_) with the laser pulse energy 40 mJ, pulse duration *τ*_*0.5*_ = 10 fs for various normalised vector potential *a*_*0*_ and waist radius *ω*_*0*_ parameters are presented Figs. 2, 3, 4 and 5 and in Tables 3 and 4. As can be seen in Fig. 2(a), on-axis normalised vector potential *a*_*0*_ is plotted beginning from the location of maximal intensity (the simulations were started near z_max_/2). Therefore, the traces display a monotonic decrease in *a*_*0*_ with propagation distance.

For the Gaussian drivers, this falloff follows the familiar diffraction-limited scaling, so the axial amplitude decays rapidly beyond the Rayleigh length. In contrast, the truncated BG drivers exhibit a much slower decay of axial amplitude because side lobes continuously replenish their central core over the extended focal zone *z*_*max*_. This reduced decay rate is the origin of the longer effective acceleration length for BG beams. Figure 2(b) shows radial cuts at the chosen axial plane. Gaussian profiles show the expected monotonic radial decay from *r* = 0, whereas for BG profiles, due to the nature of BG beams that have a central lobe plus concentric rings, the BG beams maintain a stronger on-axis driver over longer distances, whereas Gaussian beams lose axial intensity much faster due to diffraction. Moreover, the average energies *E*_*0.7E*_, *E*_*0.2E*_, standard deviation *σ*_*0.7E*_, *σ*_*0.2E*_, divergence *θ*_*0.7E*_, *θ*_*0.2E*_ and accelerated charge of electrons *q*_*0.7e*_, *q*_*0.7e*_ were calculated for the electrons having the energy > 0.7 *E*_*max*_ and > 0.2 *E*_*max*_, correspondingly. The divergences *θ*_*0.7E*_ and *θ*_*0.2E*_ were calculated as an average of *θ*_*0.7E/0.2E*_ *= arctan(u*_*x, y*_*/u*_*z*_*)* in *x* and *y* directions, where *u*_*x*_, *u*_*y*_ and *u*_*z*_ are normalised electron momenta in the directions of *x*,* y*, and *z*.

Based on the best FBPIC simulation results, the maximal energy *E*_*max*_ of electrons accelerated using the Gaussian beams (G_1_-G_5_), varies insignificantly in the range of 105–126 MeV and reaches its maximal values *E*_*max*_ of 120–126 MeV for G_4_-G_5_ beams with a moderate *a*_*0*_ value of 1.75–2.75 (Figs. 3a and 4a). It corresponds to the largest beam waist radius *w*_*0*_ of 6–7 μm, and the longest Rayleigh and dephasing and depletion distances (Table 1). The optimal plasma density for LWFA acceleration was *n*_*opt*_ = 1.3⋅10^19^ cm^− 3^, and the maximum energy values of electrons were reached at the acceleration distance of 500–750 μm.

The average energy of electrons *E*_*0.7*_ with energy of more than 0.7 *E*_*max*_ was in the range of 106–110 MeV ± 6 MeV, and the average energy of electrons *E*_*0.2*_ with energy of more than 0.2 *E*_*max*_ was 92–99 MeV ± 38 MeV. The divergence of the electron beam accelerated using G_4_-G_5_ beams was relatively small *θ*_*0.7E/0.2E*_ = 8–9 mrad, as well as the charge of accelerated electrons *q*_*0.7e/0.2e*_ = 1–3 pC. With increasing *a*_*0*_ to 3–4.1.1 and sharper focusing to *w*_*0*_ = 3–4 μm, the electron energy dispersion increased substantially, and the average energies of electrons accelerated for the G_1_-G_2_ beams dropped to *E*_*0.7*_ =85–87 MeV ± 7 MeV and *E*_*0.2*_ = 55–58 MeV ± 22 MeV, correspondingly. In fact, when the initial laser field is increased (larger *a*_*0*_), the wake grows quickly but also depletes the laser energy more rapidly. As the driver weakens, the wakefield amplitude decreases, reducing the accelerating field and making electron trapping less stable. This leads to a shorter effective acceleration length, larger energy spread, and higher beam divergence.

Thus, there is a fundamental trade-off: higher initial normalised vector potential enhances wake excitation but accelerates nonlinear evolution and pump depletion, ultimately limiting the maximum achievable electron energy. In the case of G_1_-G_2_, a the laser pulse undergoes strong nonlinear reshaping dominated by pulse front etching and pump depletion, leading to a sharp reduction of the on-axis intensity at a distance of 300–400 μm (Fig. 4b). The divergence of the electron beam and charge of accelerated electrons increased correspondingly to *θ*_*0.7E*_ = 21–27 mrad, *θ*_*0.2E*_ = 40–52 mrad, *q*_*0.7e*_ = 11–12 pC, and *q*_*0.2e*_ = 32–46 pC.

**Table 3:** Maximal energy *E*_*max*_, average energies *E*_*0.7E*_, *E*_*0.2E*_, standard deviation *σ*_*0.7E*_,* σ*_*0.2E*_, divergence *θ*_*0.7E*_, *θ*_*0.2E*_, and charge *q*_*0.7E*_, *q*_*0.2E*_, of electrons accelerated using the Gaussian beams (G_1_ - G_5_) with laser pulse energy 40 mJ, pulse duration *τ*_*0.5*_ = 10 fs, and various normalised vector potential *a*_*0*_ and waist radius *ω*_*0*_ parameters.

Beam shape	a_0_	w_0_, µm	E_max_ MeV	E_0.7E_ MeV	σ_0.7E_ MeV	E_0.2E_MeV	σ_0.2E_MeV	θ_0.7E_ mrad	θ_0.2E_ mrad	q_0.7E_ pC	q_0.2E_ pC
G_1_	4.1	3.0	105	87	7	55	22	27	52	12	46
G_2_	3.0	4.0	103	85	6	58	22	21	40	11	32
G_3_	2.5	5.0	111	94	7	77	20	14	24	11	20
G_4_	2.0	6.0	120	106	5	106	6	9	9	3	3
G_5_	1.75	7.0	126	110	6	110	6	8	8	1	1

The maximal energy *E*_*max*_ of electrons accelerated using the BG beams (BG_01_-BG_06_) varies in the range of 90–161 MeV and reaches its maximal value *E*_*max*_ of 152–161 MeV for the BG_03_-BG_04_ beams with *a*_*0max*_ = 3.3–4.1 and beam waist radius *w*_*b*_ = 7–10 μm. (Figures 3b and 5). The achieved energy of accelerated electrons was 20–27% higher than the maximal energy using G beams with the same laser pulse duration and energy.

The physical reason is that electrons remain in phase with the wake for a longer distance before dephasing, and the driver pulse maintains its amplitude longer due to the Bessel-like energy replenishment from side lobes. However, at too high _*0*_, BG beams also suffer early depletion and reduced energy gain, showing that the benefit is not infinite; there is an optimal range where extended focal length balances wake stability and dephasing. The side lobes of the BG beam continuously refuel the central lobe, effectively delaying pump depletion. This explains why the electron bunch energy and quality (lower divergence, smaller spread) are improved compared to Gaussian drivers. The optimal plasma density for LWFA acceleration was *n*_*opt*_ = 1.3 ⋅10^19^ cm^−3^. The average energy of electrons *E*_*0.7*_ was in the range of 132–137 MeV ± 12 MeV, and the average energy of electrons *E*_*0.2*_ was 132–137 MeV ± 12 MeV.

The divergence of the electron beam is *θ*_*0.7E*_ = 10–11 mrad, *θ*_*0.2E*_ = 20–25 mrad, and the charge of accelerated electrons is *q*_*0.7e*_ = 7–12 pC, and *q*_*0.2e*_ = 15–23 pC, correspondingly. With increasing *a*_*0*_ to 4.8–5.6, and sharper focusing to *ω*_*0*_ = 3–4 μm, the energy of electrons drops to *E*_*max*_= 87–90 MeV, *E*_*0.7*_ =75–82 MeV ± 7 MeV and *E*_*0.2*_ =46–49 MeV ± 22 MeV. The divergence of the electron beam and charge of accelerated electrons increased correspondingly to *θ*_*0.7E*_ = 21–27 mrad, *θ*_*0.2E*_ = 40–52 mrad, *q*_*0.7e*_ = 11–12 pC, and *q*_*0.2e*_ = 32–46 pC. The parameters of accelerated electrons using the BG_06_ beam are approximately equal to those of electrons accelerated by the G_5_ beam with *a*_*0*_ = 2.

**Table 4:** Maximal energy *E*_*max*_, average energies *E*_*0.7E*_, *E*_*0.2E*_, standard deviation *σ*_*0.7E*_,* σ*_*0.2E*_, divergence *θ*_*0.7E*_, *θ*_*0.2E*_, and charge *q*_*0.7E*_, *q*_*0.2E*_ of electrons accelerated using the Bessel-Gauss beams (BG_01_-BG_06_) with laser pulse energy 40 mJ, pulse duration *τ*_*0.5*_ = 10 fs, and various normalised vector potential *a*_*0max*_ and waist radius *ω*_*b*_ parameters.

Beam shape	a_0max_	ω_b_ µm	E_max_ MeV	E_0.7E_ MeV	σ_0.7E_MeV	E_0.2E_ MeV	σ_0.2E_MeV	θ_0.7E_ mrad	θ_0.2E_ mrad	q_0.7E_ pC	q_0.2E_ pC
BG_01_	5.4	3.0	90	75	6	46	21	25	50	11	36
BG_02_	4.7	5.0	87	82	7	49	22	30	65	15	51
BG_03_	4.1	7.0	161	137	12	99	38	10	20	7	15
BG_04_	3.3	10.0	152	132	11	100	36	11	25	12	23
BG_05_	2.7	15.0	132	115	10	103	20	12	15	12	17
BG_06_	2.1	30.0	109	96	5	96	5	7	6	1	1

### Laser pulse evolution and plasma wakefield

**Fig. 6:**
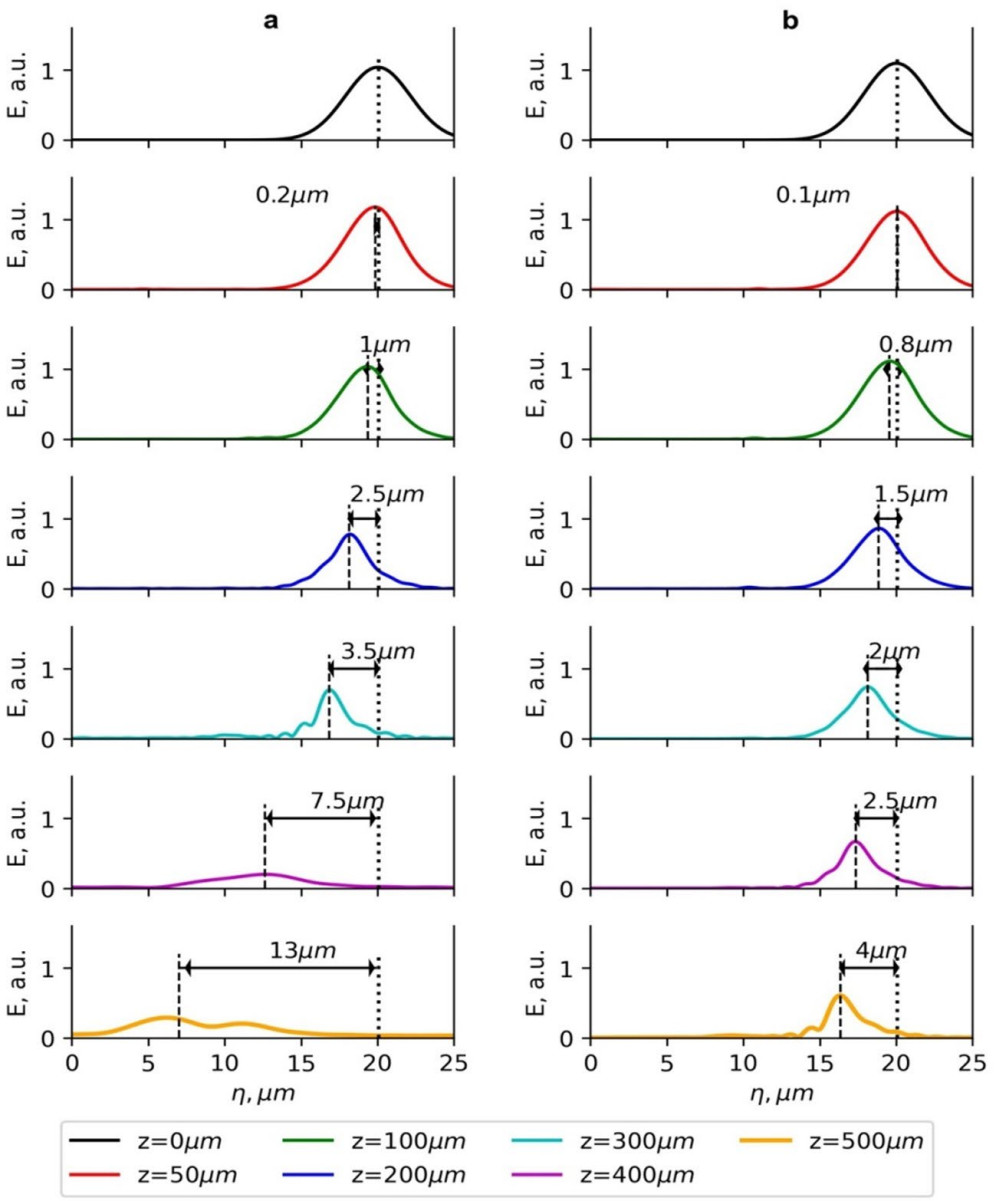
The dependence of the electric field *E* of the laser pulse of the Gaussian beam (G_2_) with normalised vector potential *a*_*0*_ =3 (**a**) and Bessel-Gauss beam (BG_04_) with normalised vector potential *a*_*0max*_=3.3 (**b**) on the longitudinal coordinate of the moving simulation window *η* for various acceleration distances *z*. The laser pulse energy is 40 mJ, and the pulse duration is *τ*_*0.5*_ = 10 fs. The plasma electron densities are 2 × 10^19^cm^− 3^ for G_2_ and 1.3 × 10^19^cm^− 3^ for BG_04_.

**Fig. 7:**
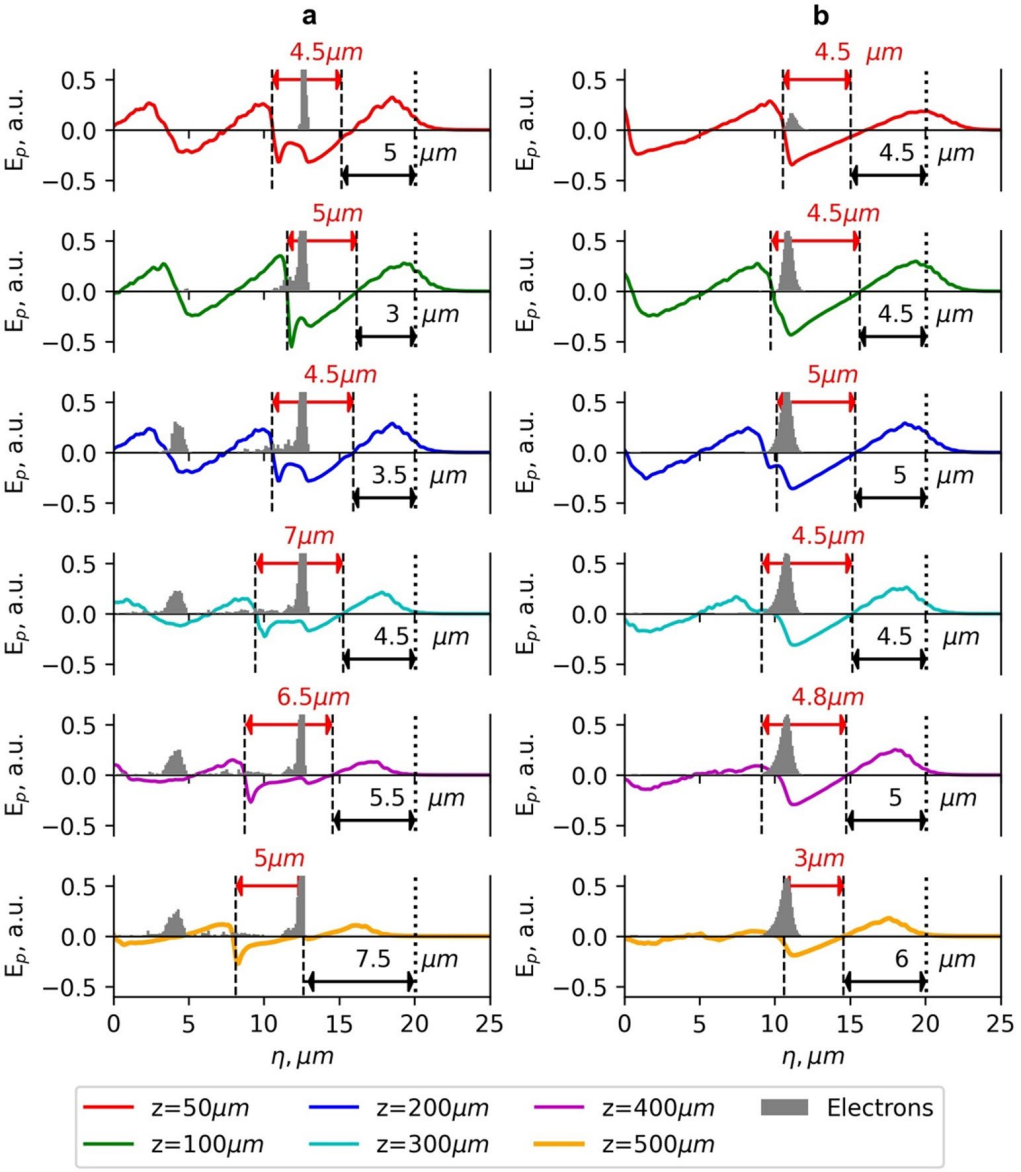
The dependence of the longitudinal plasma electric field *E*_*p*_ and position of electrons accelerated using the Gaussian beam (G_2_) with normalised vector potential *a*_*0*_ =3 (**a**) and the Bessel-Gauss beam (BG_04_) with normalised vector potential *a*_*0max*_=3 (**b**) on the longitudinal coordinate of the moving simulation window *η* for various acceleration distances *z*. The laser pulse energy is 40 mJ, and the pulse duration is *τ*_*0.5*_ = 10 fs. The plasma electron densities are 2 × 10^19^cm^− 3^ for G_2_ and 1.3 × 10^19^cm^− 3^ for BG_04_.

The best laser beam configurations for the LWFA of electrons were further analysed by investigating the effects of the laser pulse evolution and longitudinal electric field of the plasma wave. In Figs. 6 and 7, the dependence of the electric field of the laser pulse and the longitudinal plasma electric field of the G_2_ and BG_04_ beams on the longitudinal coordinate *z* is presented. The normalised vector potential *a*_*0*_ = 3–3.3.3 for both beams is similar. However, because of different optimal plasma densities, Rayleigh and dephasing lengths, the behaviour of the laser pulse evolution and the position of accelerated electrons relative to the maximum of the electric field of the pulse for *z* > 200 μm are quite different.

The maximum of the electric field of the G_2_ beam shifts to the rear of the pulse much quicker than in the BG_04_ case (Fig. 6). At the acceleration distance *z* = 300 μm, the shift is 3.5 μm for the G_2_ beam and 2 μm - for the BG_04_ beam, and at *z* > 300 μm, the G_2_ beam undergoes significant pulse evolution. At the initial distances up to ~ 50 μm, the pulse shortens, and its intensity increases due to the group velocity dispersion, resulting in a slower motion of the leading front of the pulse relative to the trailing front^47^. At the acceleration distance from 100 μm to 200 μm, the pulse intensity decreases due to the pulse front etching, and the rear front starts to form^7^.

Afterwards, the evolution of the rear pulse front due to red-shift occurs, and the losses of pulse energy increases^48^, and finally, at a distance > 300 μm, a strongly delayed trailing maximum of the laser pulse is formed (Fig. 6a). The observed evolution of the laser pulse is primarily governed by pump depletion associated with energy transfer to the plasma wake, together with longitudinal pulse reshaping and slipping arising from group-velocity differences across the pulse. However, it should be noted that these processes lead to a reduction and redistribution of the on-axis intensity during propagation; spectral red-shifting occurs as a consequence of this nonlinear evolution, but is not the dominant mechanism driving the intensity decay.

The profiles of the accelerating longitudinal electric field *E*_*p*_ for various propagation distances (Fig. 7) show that the electron energy starts to decrease when the electron beam enters the region where the plasma electric field *E*_*p*_ changes its sign to the opposite. The significant decrease in energy increase is also caused by a substantial drop in the amplitude of the accelerating field, affected by the energy losses due to the laser pulse evolution of the G_2_ beam at the acceleration distance *z* > 300 μm (Fig. 7a). The optimal density of electrons accelerated by the BG_04_ beam *n*_*opt*_ = 1.3 × 10^19^ cm^− 3^ is lower than the density *n*_*opt*_ = 1.6 × 10^19^ cm^− 3^ in the case of the G_2_ beam. The beam waist of the G_2_ beam (*w*_*0*_ = 4 μm) is smaller than the beam waist (*w*_*0*_ = 6 μm) of the illuminating G beam of the BG_04_ beam. This means the G_2_ beam is more tightly focused, which leads to stronger intensity gradients but also makes it more susceptible to dephasing and pulse evolution effects during propagation.

**Fig. 8:**
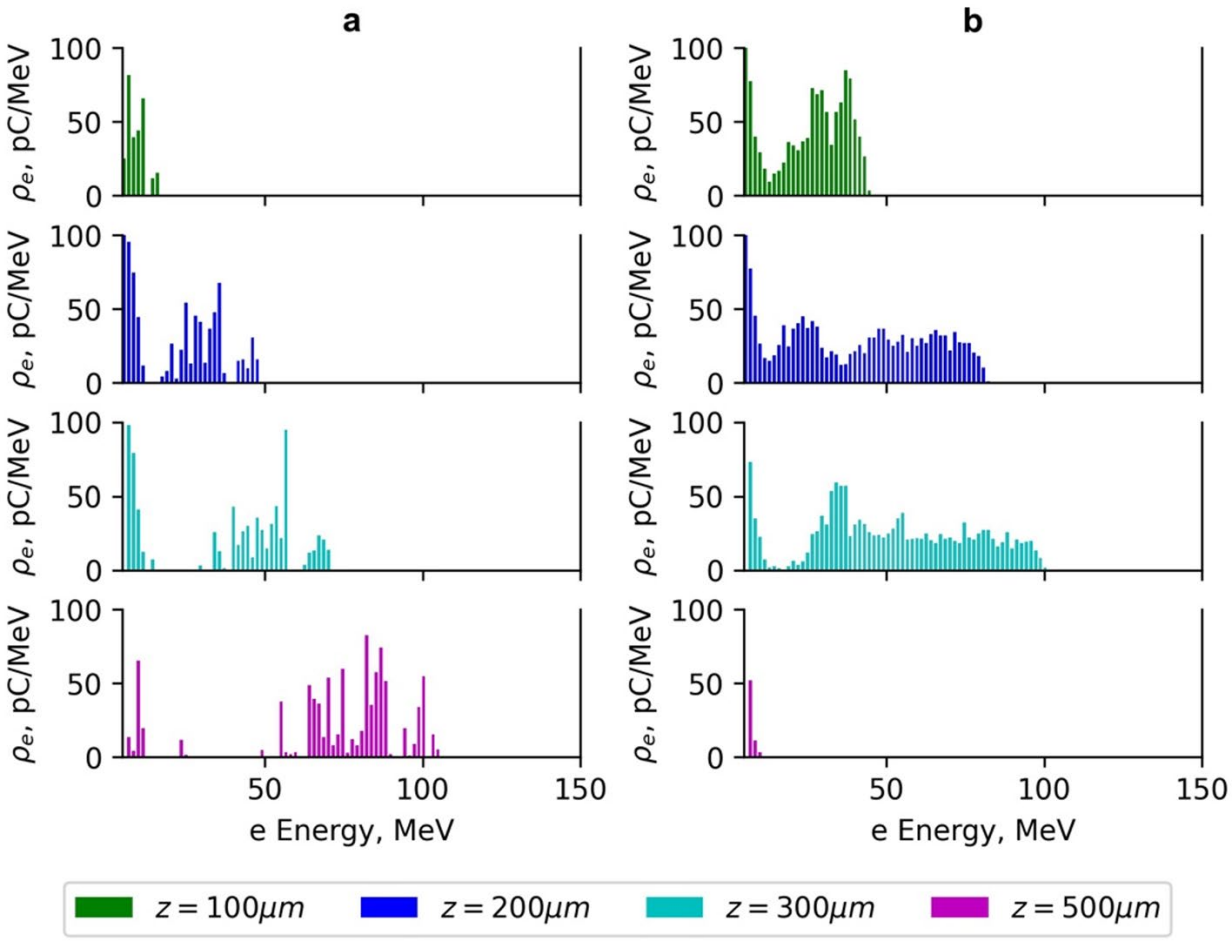
The dependence of the electron charge density per MeV on the energy of accelerated electrons using the Gaussian beam (G_4_) with normalised vector potential *a*_*0*_ =2 (**a**) and the Gaussian beam (G_2_) with normalised vector potential *a*_*0*_ =3 (**b**) on the acceleration distance *z*. The laser pulse energy is 40 mJ, pulse duration *τ*_*0.5*_ = 10 fs and the injection length of the plasma target section with 1% of N_2_ gas *L*_*N*_ =100 m.

The dephasing and pulse evolution affect the acceleration of electrons using the G_2_ beam more significantly. The intensity of the laser pulse within the 300–500 μm range drops by a factor of 2–2.5.5. limiting the maximum acceleration distance. Therefore, the energy of accelerated electrons for the BG_04_ beam is up to 27% higher compared to the acceleration using the G_2_ beam. However, when the acceleration distance exceeds 400 μm, the longitudinal plasma electric field of the BG_04_ beam decreases as well because of the broadening and intensity drop of the laser pulse due to pulse evolution effects, thereby limiting the maximum acceleration distance of electrons (Fig. 8b).

### Plasma ionisation effects

**Fig. 9:**
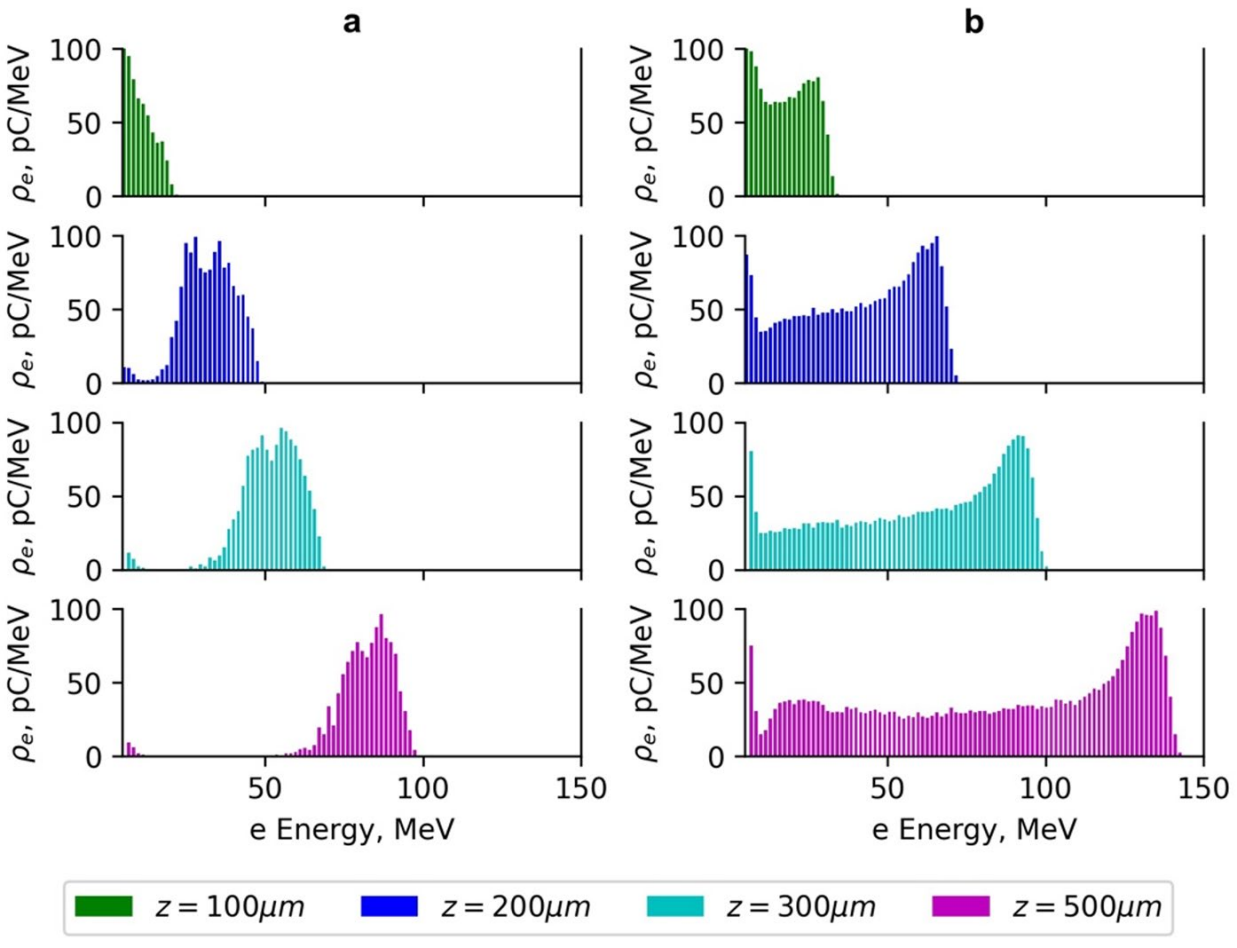
The dependence of the electron charge density per MeV on the energy of accelerated electrons using the Bessel-Gauss beam BG_06_ with normalised vector potential *a*_*0max*_ =2.1 (**a**) and Bessel-Gauss beam BG_04_ with normalised vector potential *a*_*0max*_=3.3 (**b**) on the acceleration distance *z*. The laser pulse energy is 40 mJ, pulse duration *τ*_*0.5*_ = 10 fs and the injection length of the plasma target section with 1% of N_2_ gas *L*_*N*_ =100 μm.

The analysis of the energy spectra of accelerated electrons (Figs. 8 and 9) shows that electrons form three distinct groups based on their energy: low-energy group, with electron energy less than 0.2, medium-energy group with electron energy from 0.2*E*_*max*_ to 0.7*E*_*max*_, and high -energy group with electron energy higher than 0.7*E*_*max*_. In the simulations using the normalised vector potential *a*_*0*_ = 2, the length of the plasma target section where a 1% concentration of N_2_ atoms is present was varied from 30 μm to 300 μm. For the length of =30 μm, the spectrum of high-energy electrons was relatively narrow. As the length >30 μm increased, the spectrum spread quickly, and when was more than 100 μm, the spectrum stopped changing. The injection zone length of =30 μm is hard to realise practically; therefore, in subsequent simulations, =100 μm was used.

The low-energy electron group was present in all beam configurations used in the simulation. In the medium- and high-energy regions, the electron energy spectrum depends significantly on the normalised vector potential *a*_*0*_ and plasma density *n*_*e*_. The energy spread of high-energy electrons accelerated using the Gaussian beams (G_4_-G_5_) and Bessel-Gauss beam (BG_06_) with *a*_*0*_ =1.75–2.0.75.0, remained within 30%, and the energy increased with the acceleration distance until the dephasing and depletion length of the pulse was reached (Figs. 8a and 9a).

As can be seen in Fig. 8(b), corresponding to the G beam with *a*_*0*_ = 3, a significant reduction of the visible electron charge at high energies is observed at the largest propagation distance. This effect does not indicate a loss of electrons but rather reflects a redistribution of charge toward lower energies. At a higher *a*_*0*_, the laser pulse undergoes stronger self-modulation and pulse front etching, leading to a rapid decrease in the on-axis laser intensity beyond z ≈ 300 μm. As a result, the accelerating wakefield weakens and, finally, reverses its phase, causing previously accelerated electrons to decelerate and shift to lower energies. Since Fig. 8 shows the charge density within a fixed energy range, this redistribution appears as a reduction of charge in the high-energy part of the spectrum. It should be noted that Fig. 8(a) and Fig. 8(b) correspond to different normalised vector potentials (*a*_*0*_ = 2 and *a*_*0*_ = 3) and therefore represent different acceleration regimes. The figure is intended to illustrate the spectral evolution with propagation distance for each case individually rather than to provide a direct quantitative comparison between different *a*_*0*_ values.

Such dependence of the width of the energy spectrum is determined by the dependence of the intensity of G pulses on the propagation distance (Figs. 4b and 5b). For the propagation distance >300 μm, the laser pulse strength parameter _*0*_ drops below 1.8 value, and is smaller than the ionisation threshold of nitrogen 6th (K-shell) electron^48^. Therefore, the generation of the new electrons in the plasma target section, where 1% density of the *N*_2_ atoms are present, even for =300 m, is truncated, and the energy spectrum remains quasi-monoenergetic. Due to the self-injection, the energy spread of high-energy electrons accelerated by the Gaussian beams (G_1_-G_3_) and Bessel-Gauss beams (BG_01_-BG_05_) with higher _*0*_, in the range 2.5–5.6, has increased.

Figure 8b − 9b show the energy spectra of accelerated electrons using G_2_ and BG_04_ with normalised vector potential *a*_*0*_ = 3–3.3.3. The maximal energy of electrons using the beam G_2_ was reached at the distance of *z* = 300 μm. The average energy *E*_*0.7*_ was 103 MeV ± 6 MeV. Due to the higher optimal plasma density and shorter dephasing length of 295 μm of the G_2_ beam relative to the 379 μm of the BG_04_ beam, the average energy of electrons accelerated *E*_*0.7*_ using the G_2_ beam was smaller than that of the BG_04_ beam. The spread interval of the medium-energy electron group increased with the acceleration distance from *z* = 100 μm to *z* = 300 μm (Fig. 8b − 9b). The electrons accelerated using the beam G_2_ were decelerated at a distance of *z* = 500 μm because of the change in the sign of the longitudinal plasma electric field to the opposite (Fig. 8b).

For the beam BG_04_, a high-energy electron zone with an average energy *E*_*0.7*_ of 132 MeV ± 11 MeV was observed. The energy spread in the high-energy zone was 30%, and the maximal energy was reached at the acceleration distance of *z* = 500 μm (Fig. 9b). The medium-energy electron group with energy of 30–120 MeV was also present. The spread interval of the medium-energy electron group increased with the acceleration distance from *z* = 100 μm to *z* = 500 μm.

## Conclusions

This paper demonstrates, through FBPIC simulations, that BG beams can significantly enhance LWFA performance compared to conventional G beams when driven by 40 mJ, 10 fs laser pulses. The key advantage of BG beams arises from their extended focal zone and side-lobe energy replenishment, which sustain higher on-axis intensity over longer propagation distances. This mitigates divergence and pump depletion, and allows electrons to remain in the accelerating phase of the wakefield beyond the Rayleigh limit for a longer distance, resulting in a 20–27% increase in maximum electron energy, reaching 150–160 MeV.

At moderate normalised vector potential (*a*_*0*_ = 1.75–2.0), ionisation injection produces quasi-monoenergetic spectra with energy spreads below 30%, whereas higher *a*_*0*_ values lead to stronger self-injection and increased divergence. Laser pulse evolution and pump depletion remain the main limitations, with pulse intensity decreasing by a factor of 2–2.5 after 300–500 μm of propagation. This restricts further energy gain even in the BG case. Nonetheless, the ability of BG beams to maintain guiding and delay diffraction provides a clear pathway to improving LWFA at sub-100 mJ laser energies.

These results indicate that BG-driven LWFA provides a promising approach for improving electron acceleration in compact, high-repetition-rate systems operating at sub-100 mJ energies. The demonstrated improvements in energy gain, charge, and stability are particularly relevant for compact radiation sources, advanced accelerator concepts, and applied plasma-based technologies.

## Data Availability

All data supporting the findings of this research are contained within the article.

## Abbreviations

PIC: Particle-In-Cell
FBPIC: Fourier–Bessel Particle-In-Cell
G: Gaussian
BG: Bessel-Gauss
LWFA: Laser Wakefield Acceleration
L_d_: Dephasing Length
L_dep_: Pump Depletion Length
L_acc_: Acceleration Lengths
STII: Self-Truncated Ionisation Injection
FWHM: Full Width Half Maximum
